# The κ-opioid receptor-induced autophagy is implicated in stress-driven synaptic alterations

**DOI:** 10.3389/fnmol.2022.1039135

**Published:** 2022-11-16

**Authors:** Christos Karoussiotis, Aggeliki Sotiriou, Alexia Polissidis, Alexandra Symeonof, Danae Papavranoussi-Daponte, Vassiliki Nikoletopoulou, Zafiroula Georgoussi

**Affiliations:** ^1^Laboratory of Cellular Signaling and Molecular Pharmacology, Institute of Biosciences and Applications, National Centre for Scientific Research “Demokritos”, Athens, Greece; ^2^Institute of Molecular Biology and Biotechnology, Foundation for Research and Technology-Hellas, Heraklion, Crete, Greece; ^3^Center for Clinical Research, Experimental Surgery, and Translational Research, Biomedical Research Foundation of the Academy of Athens, Athens, Greece; ^4^Département des Neurosciences Fondamentales, University of Lausanne, Lausanne, Switzerland

**Keywords:** κ-opioid receptor, autophagy, Beclin 1, ERK1,2, Gi/o, CREB, dynorphin, synaptic alterations

## Abstract

Recent evidence has shown that G protein-coupled receptors (GPCRs) are direct sensors of the autophagic machinery and opioid receptors regulate neuronal plasticity and neurotransmission with an as yet unclarified mechanism. Using *in vitro* and *in vivo* experimental approaches, this study aims to clarify the potential role of autophagy and κ-opioid receptor (κ-OR) signaling in synaptic alterations. We hereby demonstrate that the selective κ-OR agonist U50,488H, induces autophagy in a time-and dose-dependent manner in Neuro-2A cells stably expressing the human κ-OR by upregulating microtubule-associated protein Light Chain 3-II (LC3-II), Beclin 1 and Autophagy Related Gene 5 (ATG5). Pretreatment of neuronal cells with pertussis toxin blocked the above κ-OR-mediated cellular responses. Our molecular analysis also revealed a κ-OR-driven upregulation of *becn1* gene through ERK1,2-dependent activation of the transcription factor CREB in Neuro-2A cells. Moreover, our studies demonstrated that sub-chronic U50,488H administration in mice causes profound increases of specific autophagic markers in the hippocampus with a concomitant decrease of several pre-and post-synaptic proteins, such as spinophilin, postsynaptic density protein 95 (PSD-95) and synaptosomal associated protein 25 (SNAP25). Finally, using acute stress, a stimulus known to increase the levels of the endogenous κ-OR ligand dynorphin, we are demonstrating that administration of the κ-ΟR selective antagonist, nor-binaltorphimine (norBNI), blocks the induction of autophagy and the stress-evoked reduction of synaptic proteins in the hippocampus. These findings provide novel insights about the essential role of autophagic machinery into the mechanisms through which κ-OR signaling regulates brain plasticity.

## Introduction

The κ-opioid receptor (κ-OR), that is distributed in the central and peripheral nervous system mediates the diverse effects of opioids ranging from pain perception, neurotransmitter release and respiratory depression to the regulation of a variety of psychiatric disorders including anxiety and addiction (Bruchas et al., 2010). The κ-OR and its endogenous neuropeptide, dynorphin A, were found to play a key role in modulating anxiety and stress-related behaviors. Thus, stress has been shown to increase endogenous dynorphin levels and up-regulate κ-OR signaling in the nucleus accumbens and the CA3 region of the hippocampus (Shirayama et al., 2004). Ablation of κ-OR from brain dopaminergic neurons produced anxiolytic effects, confirming that the regulation of dopaminergic neurotransmission by κ-OR is critical for manifestation of stress and anxiety (Van’t Veer and Carlezon, 2013).

Recent results also suggest that κ-ΟR antagonists possess promising antidepressant potential, indicating that the κ-ΟR and its endogenous neuropeptide ligand, dynorphin A, are critical mediators of stress and mood disorders with specific κ-ΟR antagonists being currently tested in phase II clinical trials (Lutz and Kieffer, 2013; Rorick-Kehn et al., 2014; Hang et al., 2015). However, the signaling constituents responsible for the neurobiological responses that regulate these physiological phenomena have yet to be deduced.

In the brain, the κ-OR is coupled to pertussis toxin sensitive Gi/o proteins to regulate a variety of downstream effectors including adenylyl cyclase, K^+^ and Ca^2+^ channels, phospholipase C, and ERK1,2 phosphorylation (Schulz et al., 2004; Bruchas et al., 2010). Such diverse signaling events are mediated not only by interactions with G proteins but also by other proteins that determine the generated signal and alterations in the trafficking, targeting and fine tuning of this receptor (Bruchas et al., 2010; Georgoussi et al., 2012; Papakonstantinou et al., 2015).

Macroautophagy, herein referred as autophagy, is a highly conserved degradation process in which proteins and organelles are engulfed in autophagic vesicles and subsequently targeted for degradation in lysosomes (Chen and Klionsky, 2011). Autophagy plays an important role in many organisms upon exposure to stress but is also considered to be an important physiological mechanism in neuronal homeostasis. In neurons, autophagy occurs constitutively under physiological conditions, while impaired autophagy is implicated in many neurodevelopmental and neurodegenerative disorders (Nikoletopoulou et al., 2015). Recent evidence suggests that autophagy regulates the development and function of axons, dendrites and synapses, whereas aberrations in neuronal autophagy contribute to pathological changes. Autophagy alters the kinetics of neurotransmitter release and the density of synaptic vesicles and is also implicated in the degradation of postsynaptic receptors such as the GABA_A_ and AMPA receptors (Hernandez et al., 2012; Nikoletopoulou et al., 2015). Autophagy contributes to such alterations by degrading specific synaptic proteins involved in spine remodeling and retraction suggesting a direct link between autophagy and pruning of synaptic connections. Consequently, the targeting of neuronal autophagy may have great clinical implications in terms of treatment of various psychiatric disorders (Shen et al., 2015; Nikoletopoulou et al., 2017).

Additional findings also suggest that GPCRs are direct regulators of autophagy (Wauson et al., 2014). Previous studies have shown that exposure of SH-SY5Y and endothelial cells to morphine or methamphetamine respectively, induces autophagy through the involvement of opioid receptors by as yet undefined mechanisms (Zhao et al., 2010; Ma et al., 2014). Moreover, other studies have shown that morphine dysregulates synaptic balance in the hippocampus via a novel signaling pathway involving reactive oxygen species, endoplasmic reticulum stress and autophagy (Cai et al., 2016). Although opioid receptors and interacting, Gi/o and Regulators of G protein signaling (RGS) proteins, were shown to play key roles in neuronal signaling (Georganta et al., 2013; Papakonstantinou et al., 2015; Pallaki et al., 2017), it is unknown whether κ-OR activation by specific agonists can induce the autophagic machinery in neuroblastoma cells stably expressing the human κ-OR and whether these effects could result in synaptic alterations implicated in stress-related behaviors.

The present study demonstrates a novel signaling pathway via which a specific representative of opioid receptors, κ-ΟR, induces autophagy resulting in synaptosomal changes. In addition, we show that administration of the κ-OR specific antagonist, nor-BNI, to mice during acute stress exposure [daily forced swim test (FST)], prevents autophagy induction and stress-induced degradation of synaptic proteins. These results provide a novel insight to the role of this receptor in the regulation of neuronal autophagy and demonstrate that κ-ΟR-mediated autophagy is responsible for specific changes in stress-induced synaptic alterations.

## Materials and methods

### Reagents

Opioid ligands U50,488H, naloxone and nor-BNI (nor-binaltorphimine) were purchased from Tocris Bioscience (Cookson MI, United States). Dynorphin_1-13_, pertussis toxin, bafilomycin A1, phosphatase inhibitors and TRI-reagent for RNA extraction were from Sigma Aldrich (St Louis, MO, United States). Protease inhibitors were from Roche (Roche Diagnostics, Basel, Switzerland). Protein A/G agarose beads and PD98059 (MEKI inhibitor SC-3532) were from Santa Cruz Biotechnology (Santa Cruz, CA, United States). Kapa Hi-Fi PCR, Kapa SYBR Fast QPCR kits for ChIP assay and Real-time PCR, respectively, were purchased from Kapa Biosystems (Roche, IN, United States). All reagents were purchased from Sigma Aldrich (Sigma Aldrich MI, United States).

### Antibodies

Antibodies used for immunoblotting and microscopy analysis were the following: LC3B (Cell Signaling Technology Cat# 2775, RRID:AB_915950), p-CREB (Cell Signaling Technology Cat# 9198, RRID:AB_2561044), CREB (Cell Signaling Technology Cat# 9197, RRID:AB_331277), p-ERK1,2 (Cell Signaling Technology Cat# 9101, RRID:AB_331646), ERK1,2 (Cell Signaling Technology Cat# 9102, RRID:AB_330744), β-actin (Cell Signaling Technology Cat# 8457, RRID:AB_10950489), β-tubulin (Cell Signaling Technology Cat# 2128, RRID:AB_823664), p-SAPK/JNK (Cell Signaling Technology Cat# 9251, RRID:AB_331659) ULK1 (Cell Signaling Technology Cat# 8054, RRID:AB_11178668), FIP200 (Cell Signaling Technology Cat# 12436, RRID:AB_2797913) and β-ΙΙΙ tubulin (Cell Signaling Technology Cat# 5568, RRID:AB_10694505) were purchased from Cell Signaling Technology Inc. (Danvers, MA, United States). Beclin1 (Santa Cruz Biotechnology Cat# sc-11,427, RRID:AB_2064465), p62 (Santa Cruz Biotechnology Cat# sc-48,402, RRID:AB_2255371), ATG5 (Santa Cruz Biotechnology Cat# sc-133,158, RRID:AB_2243288), Neurabin II/Spinophilin (Santa Cruz Biotechnology Cat# sc-14,774, RRID:AB_2169477) PSD-95 (Santa Cruz Biotechnology Cat# sc-32,290, RRID:AB_628114) and SNAP25 (Santa Cruz Biotechnology Cat# sc-20,038, RRID:AB-628264) antibodies were purchased from Santa Cruz Biotechnology (Santa Cruz, CA, United States). All secondary antibodies were from KPL (Maryland, United States). For confocal microscopy the anti-rabbit Alexa-Fluor 568 (Thermo Fisher Scientific Cat# A^−11,011^, RRID: AB_143157) and anti-mouse CFL 488 (Santa Cruz Biotechnology Cat# sc-362,257, RRID:AB_10989084) sera were used.

### Cell cultures

Neuro-2A neuroblastoma cells (ATCC Cat# CCL-131, RRID: CVCL_0470) stably expressing the myc-tagged human κ-OR (κ-Νeuro-2A) were cultured in Dulbecco’s modified Eagle’s medium (Merck Millipore, MA. United States) with 2 mM L-glutamine, 100 U/mL penicillin, 100 μg/mL streptomycin and 10% fetal bovine serum (Biosera, France) under humidified atmosphere 5% CO_2_ at 37°C. For the generation of the stable cell line expressing the human myc-κ-OR, Neuro-2A cells were transfected with the hκ-ΟR in pA3M vector (κ-Νeuro-2Α). Clonal cell lines stably expressing the κ-ΟR (260 fmol/mg protein) were established upon selection with G418. The expression levels of κ-OR were determined by [^3^H]-diprenorphine saturation binding of cell membranes, as described by Morou and Georgoussi (2005), and western blotting. For the pertussis toxin (PTX) ribosylation experiments, κ-Neuro-2A cells were treated with PTX (100 ng/mL) for 16 h prior of agonist stimulation as described by Georganta et al. (2013).

### Animals and treatments

Animal maintenance and experimentation were conducted in strict compliance with the European and National Law for Laboratory Animal use (Directive 2010/63/EU and Greek Law 161/91), the FELASA recommendations and the ethical and practical guidelines for the care and use of laboratory animals set by the competent veterinary services of Athens. All experiments were carried out in wild-type C57BL/6 J mice. Three-month-old male mice were divided into two groups (*n* = 4/ group) and injected intraperitoneally once per day with saline or 5 mg/kg U50,488H for 6 consecutive days. The mice were sacrificed 3 h after the last U50,488H/saline injection and the hippocampus, cortex, and striatum were rapidly dissected on ice. Isolated regions were placed in cold PBS and immediately homogenized by sonication in RIPA buffer containing 50 mM Tris–HCl pH 7.2, 150 mM NaCl, 2 mM EDTA, 1% Triton X-100, 1% sodium deoxycholate, 0.5% SDS and 1 mM dithiothreitol in the presence of protease inhibitors and incubated for 1 h at 4 ^ο^C. The resulting supernatant was collected after centrifugation at 8,000 x g for 20 min.

### Primary neuronal cultures

Hippocampi were isolated on embryonic day 16 (E16.5) rinsed and dissected in ice-cold PBS and incubated with 0.25% trypsin for 25 min at 37°C. The digestion was terminated by the addition of DMEM solution supplemented with 10% FBS followed by trituration tissue dissociation. The resulting cells were centrifuged for 5 min at 800 rpm and neurons dissolved in Neurobasal medium supplemented with 2% B-27, 0.5 mM L-glutamine and 1% penicillin/streptomycin. The cells were plated at a density of 2×10^5^ cells/well in 6-well poly-L-lysine-coated tissue culture dishes or on coverslips where necessary. Cells were cultured for 10 days (DIV10) for neuron maturation under 5% CO_2_ at 37°C. Neuronal purity was >90% as determined by immunofluorescence using the neuronal marker βIII-tubulin.

### Isolation of synaptosomes

Synaptosomes were isolated as previously described by Carlin et al. (1980). Briefly, mice at postnatal days 90–95 were treated as described above. Brain hippocampi from the two animal groups were collected, rinsed and homogenized in solution A, consisting of 0.32 M Sucrose, 1 mM NaHCO_3_, 1 mM MgCl_2_, 0.5 mM CaCl_2_•H2O, 10 mM sodium pyrophosphate and protease inhibitors using a dounce homogenizer. After centrifugation at 1,400 × g for 10 min at 4°C, the resulting supernatants were kept and the pellets were diluted 10% w/v in solution A and spun at 710xg for 10 min. The supernatants were collected and centrifuged at 13,800 × g for 10 min at 4°C. The pellets were resuspended in 0.32 M sucrose and 1 mM NaHCO_3_ using a dounce homogenizer and layered on a discontinuous sucrose gradient (10 mL-layers of 1.2 M, 1.0 M and 0.85 M sucrose). After centrifugation at 82,500 × g for 2 h, the synaptosomes from U50,488H-or saline-injected mice were isolated from the 1.2–1 M sucrose layer.

### Western blotting

Neuronal cells treated or not with different κ-OR ligands were rinsed in PBS containing 0.1 mM PMSF and 0.1 mM Na_3_VO_4_. Cells were lysed in buffer containing 25 mM Tris pH 7.4, 150 mM NaCl, 5 mM EDTA, 1% Igepal, 1 mM dithiothreitol and 1% of a protease and phosphatase inhibitor cocktail. Proteins were separated on 10 or 17%-SDS-PAGE and transferred onto PVDF membranes (Immobilon-P, Merck Millipore, ΜΑ, United States) as described by Papakonstantinou et al. (2015). Blots were visualized using enhanced chemiluminescence (Pierce-Thermo Scientific, MA, United States) and a luminescent image analyzer (Fujifilm LAS-4000). The densitometric analyses were performed using ImageJ software (National Institute of Health, Bethesda, MD, United States). β-Actin and β-tubulin were used as loading controls for protein analysis.

### Detection of mitogen activated protein kinases phosphorylation

κ-Neuro-2A cells were cultured in 60 mm plates for 48 h in the presence or absence of U50,488H (20 μΜ) for 15 min and 6 h at 37°C. Cell monolayers were rinsed with PBS following the procedure as described by Fourla et al. (2012). Where necessary cells were exposed to the ERK1, 2 inhibitor PD98059 (20 μΜ for 45 min), or the JNK inhibitor SP600125 (20 μΜ for 30 min) prior to agonist treatment. The proteins were resolved in 10% SDS-PAGE and visualized by immunoblotting with the appropriate antibodies as described previously by Morou and Georgoussi (2005).

### Immunofluoresence staining

Primary neuronal cultures on poly-L-lysine coated coverslips were treated with κ-ΟR ligands for different time intervals. Cells were fixed for 10 min with 100% methanol at −20°C and incubated overnight at 4°C with the anti-LC3B antibody (1:200) followed by 2 h incubation with the fluorescein-conjugated secondary antibody Alexa fluor 488 goat anti-rabbit (1:100) and TO-PRO-3 (1:500) for nuclear staining. The cells were mounted on slides with Vectashield mounting media (Vector Laboratories Inc., Burlingame, CA, United States) and visualized using a Leica SP8 confocal microscope (Leica Microsystems, Germany).

### Measurements of branching in hippocampal neurons

Primary hippocampal neurons were isolated from mice embryo at E16.5 and coated at poly-L-lysine coverslips with Neurobasal medium supplemented with 2% B-27, 0.5 mM L-glutamine and 1% penicillin/streptomycin. The cells were plated at a density of 2×10^5^ cells/well in 6-well tissue culture dishes. Cells were cultured for 10 days (DIV10) for neuron maturation under 5% CO_2_ at 37°C. Then the cells were treated or not with 20 μM U50,488H for 24 h and subsequently immunolabeled with the neuronal marker βIII-tubulin (TUJ1, 1:1000) to visualize the neurites and with TO-PRO-3 (1:500) for nuclear staining. The cells were mounted on slides and visualized with confocal microscopy. To count the number of branches 100 cells per condition were measured from 3 independent experiments. The branching of neurons was estimated by counting the branches that arise from each neurite using the Image J software..

### Co-immunoprecipitation assay

Hippocampi from wild-type C57BL/6 J mice were isolated and lysed in RIPA lysis buffer containing 1% v/v Triton X-100, 0.2% w/v SDS, 1% w/v sodium deoxycholate, 50 mM Tris–HCl (pH 7.6), 5 mM EDTA, 150 mM NaCl, 50 mM NaF, supplemented with antipain, leupeptin, benzamidine (1 μg/mL each), complete EDTA-free inhibitors, 1 mM PMSF and 1 mM sodium orthovanadate. Approximately, 800 μg of the clarified cell lysates were incubated with an LC3B monoclonal antibody (2 μg) overnight at 4°C. Normal rabbit serum (NRS) was used as control. Immune complexes were recovered on protein A/G agarose beads for 3 h at 4°C, washed extensively with buffer consisted of 25 mM Tris–HCl (pH 7.4), 300 mM NaCl, 1 mM EGTA, 1 mM EDTA, 1% Triton X-100, 0.2 mM PMSF and 0.2 mM Na_3_VO_4_, subjected to SDS-PAGE and transferred onto polyvinylidene (PVDF) membranes. Co-immunoprecipitation of cell lysate proteins was verified by immunoblotting using the appropriate antibodies.

### Chromatin immunoprecipitation

κ-Neuro-2A cells treated or not with U50,488H for 6 h were cross-linked with 1% formaldehyde for 10 min at room temperature followed by 5 min incubation with 0.125 mM glycine as previously described (Carey et al., 2009). Briefly, isolated nuclei were sonicated and the extracted chromatin (200 μg) supplemented with protease inhibitors was immunoprecipitated using a ChIP grade antibody against CREB or NRS. The crosslinked protein complexes were incubated for 2 h at 4°C under rotation with pre-blocked salmon sperm DNA and 5% BSA protein A/G agarose beads. Following extensive washes with 0.1% SDS, 1% Triton X-100, 2 mM EDTA, 150 mM NaCl, 20 mM Tris-HCl pH 7.5, the immune complexes were incubated overnight in 1% SDS, 100 mM NaHCO3 and proteinase K. The immunoprecipitated DNA was extracted by phenol-chloroform-isoamylyl alcohol and PCR was carried out using the following primers for Beclin1, *BECN1* (forward) 5’-CGGGTAAACAGGGATCTGGAG-3′ and (reverse) 5’-GCCAGGGACTCTAGGCTTCTT-3′, spanning the putative CRE binding site in the mouse *Becn1* promoter. The PCR products were separated on 2% agarose gels.

### RNA extraction and real-time polymerase chain reaction

Total RNA was extracted with TRI-reagent from control or U50,488H treated κ-Neuro-2A cells according to manufacturer’s instructions. Total RNA (1 μg) was used as template for cDNA synthesis using SuperScript II reverse transcriptase (Thermo Fisher Scientific). The following primers were designed for Real Time-PCR: *Becn1*, (forward):5′-GGCCAATAAGATGGGTCTGA-3′; (reverse) 5′-GCTGCACACAGTCCAGAAAA-3′; for *ATG5*, 5′ (forward) AAGTCTGTCC-TTCCGCAGTC-3′; (reverse) GAAGAAAGTTATCTGGGTAGCTCA-3′; for *GAPDH (*forward) 5’-TGTGTCCGTCGTGGATCTGA-3′, (reverse) 5’-CCTGCTTCACCACCTTCTTGA-3′, using a ΜΧ3000P QPCR System (Stratagene, La Jolla, CA, United States). The expression of the mRNAs was calculated using the ΔCt method.

### Forced swim test

The forced swim test (FST) was adapted from McLaughlin et al. (2003) with minor modifications. Briefly mice were divided into 4 groups (*n* = 6/group) and on day 1, they were injected intraperitoneally with the κ-ΟR antagonist, nor-BNI (10 mg/kg), or vehicle and placed in a 5 l beaker (40 cm tall × 25 cm in diameter) filled with 2.5 l of 30°C water for a single swim trial of 15 min. On day 2, the animals were subjected to 4x FST trials, 6 min long, with 6 min intervals. During the last 4 min of the trial mice were recorded and the time spent immobile was counted as “stressed behavior.” After each trial, the mouse was removed from the water, dried with towels and returned to its home cage for at least 6 min before further testing. Immobility was defined as the animal remaining motionless or making only minor non-escape-related movements. To qualify as immobility each posture must be clearly visible and sustained for a minimum of 2 s. Immobility was measured with the specialized video tracking software Ethovision XT9.0 (Noldus, Netherlands). Difficulty in swimming or staying afloat were criteria for exclusion, however, no mice met these criteria in this study.

### Statistical analysis

Statistical analysis was performed using one or two-way analysis of variance (ANOVA) following by Tukey’s *t* test for *post-hoc* comparisons. All experiments were repeated at least three times. Bands were quantified by densitometric analysis using Image J software (National Institute of Health, Bethesda, MD, United States) and expressed as mean ± SEM. Representative experiments are shown and statistical significance is shown in each figure legend.

## Results

### Selective κ-opioid receptor agonists induce the autophagic flux in neuronal cells

**Figure 1:**
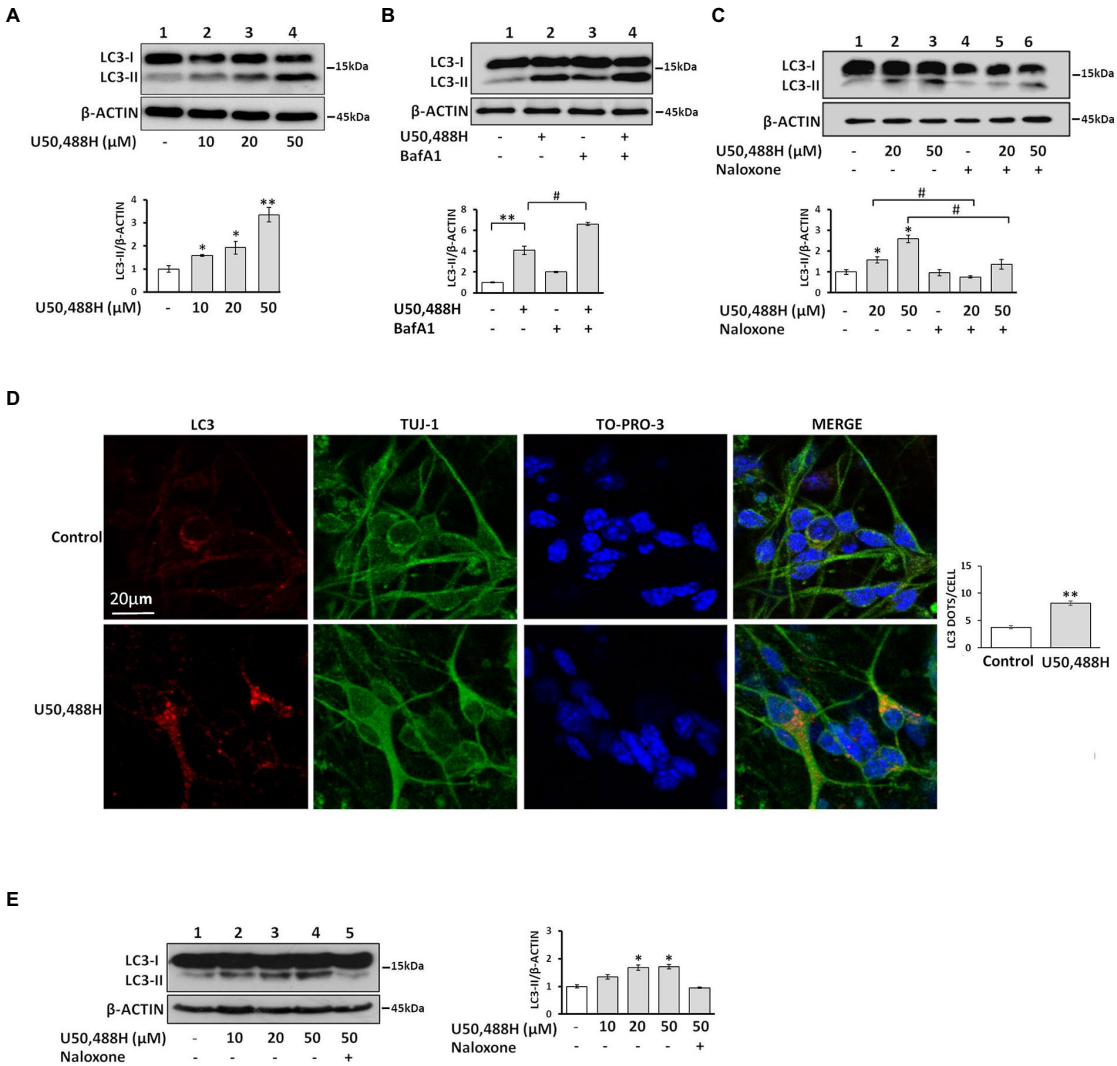
Activation of the κ-OR induces the autophagic machinery in neuronal cells. **(A)** Treatment of κ-Neuro-2A cells with the κ-OR agonist U50,488H results in upregulation of LC3-II in a dose-dependent manner. **(B)** κ-Neuro-2A cells were pre-treated with 20 nM BafA1 for 16 h, prior to U50,488H (20 μΜ), or **(C)**, with 50 μM naloxone for 45 min, prior to agonist exposure for 6 h. All cell lysates (50 μg) were subjected to 17% SDS-PAGE for western immunoblotting to detect the autophagosome formation, top panels, immunoblots of LC3-II and β-actin; bottom panels, quantification of LC3-II (measured with an anti-LC3B antibody and normalized to β-actin levels). **(D)** Primary hippocampal cultures were exposed to U50,488H (20 μΜ) for 6 h. Confocal images of 10 DIV embryonic hippocampal neurons, immunostained with an anti-LC3B antibody (1:200) to label autophagosomes, Tuj-1 (1:1000) to label dendrites, and the nuclear dye TO-PRO-3 (1:500). **(E)** LC3-II is up-regulated dose dependently upon U50,488H exposure (6 h) in embryonic primary neuronal cultures (lanes 2–4). Lane 5, represents lysates of neuronal cultures treated with 50 μΜ naloxone prior to U50,488H administration. Top panel represents immunoblots of LC3-II and β-actin. Quantification of LC3-II was normalized to β-actin levels. All experiments were performed independently at least 3 times. Error bars represent mean values ± SEM. Statistical analysis was performed using one or two-way ANOVA. *p < 0.05 and **p < 0.01 as compared in the absence of agonist, ^#^*p* < 0.05 as compared with samples in the presence of U50,488H.

It is known that neuronal autophagy is involved in cell growth, survival and synaptic plasticity (Nikoletopoulou et al., 2015, 2017). Here, we investigated whether specific κ-OR agonists could trigger autophagy in κ-Νeuro-2A cells and modulate synaptic organization. We thus treated Neuro-2A cells, stably expressing κ-ΟR, with U50,488H, a κ-OR-specific agonist, and monitored the levels of the lipidated LC3 (LC3-II), a reliable and specific marker of autophagosome formation located at the membrane of the autophagosome. As shown in Figure 1A, increasing concentrations of U50,488H for 6 h caused a dose-dependent increase in LC3-II accumulation. Addition of the lysosomal inhibitor bafilomycin A1 (BafA1), which prevents the fusion of autophagosomes with lysosomes indicated a significant increase in LC3-II levels in cells exposed simultaneously to U50,488H and BafA1, relative to BafA1 alone (Figure 1B), indicating that κ-OR activation upregulates the autophagic flux. Finally, LC3-II accumulation was markedly reversed upon treatment with the opioid antagonist, naloxone, prior to U50,488H exposure, further confirming the κ-ΟR-dependent autophagic activation (Figure 1C).

To recapitulate U50,488H-mediated autophagy in a native neuronal milieu, we treated hippocampal neuronal cultures to U50,488H, that resulted in a significant increase of LC3-positive autophagosomes that appeared as puncta, compared with untreated controls (Figure 1D). Consistent with these findings, immunoblot analysis of primary hippocampal cultures showed that naloxone blocked the increase in LC3-II accumulation caused by U50,488H exposure (Figure 1E). Collectively, these results demonstrate that κ-OR activation induces autophagy in neuronal cells.

**Figure 2:**
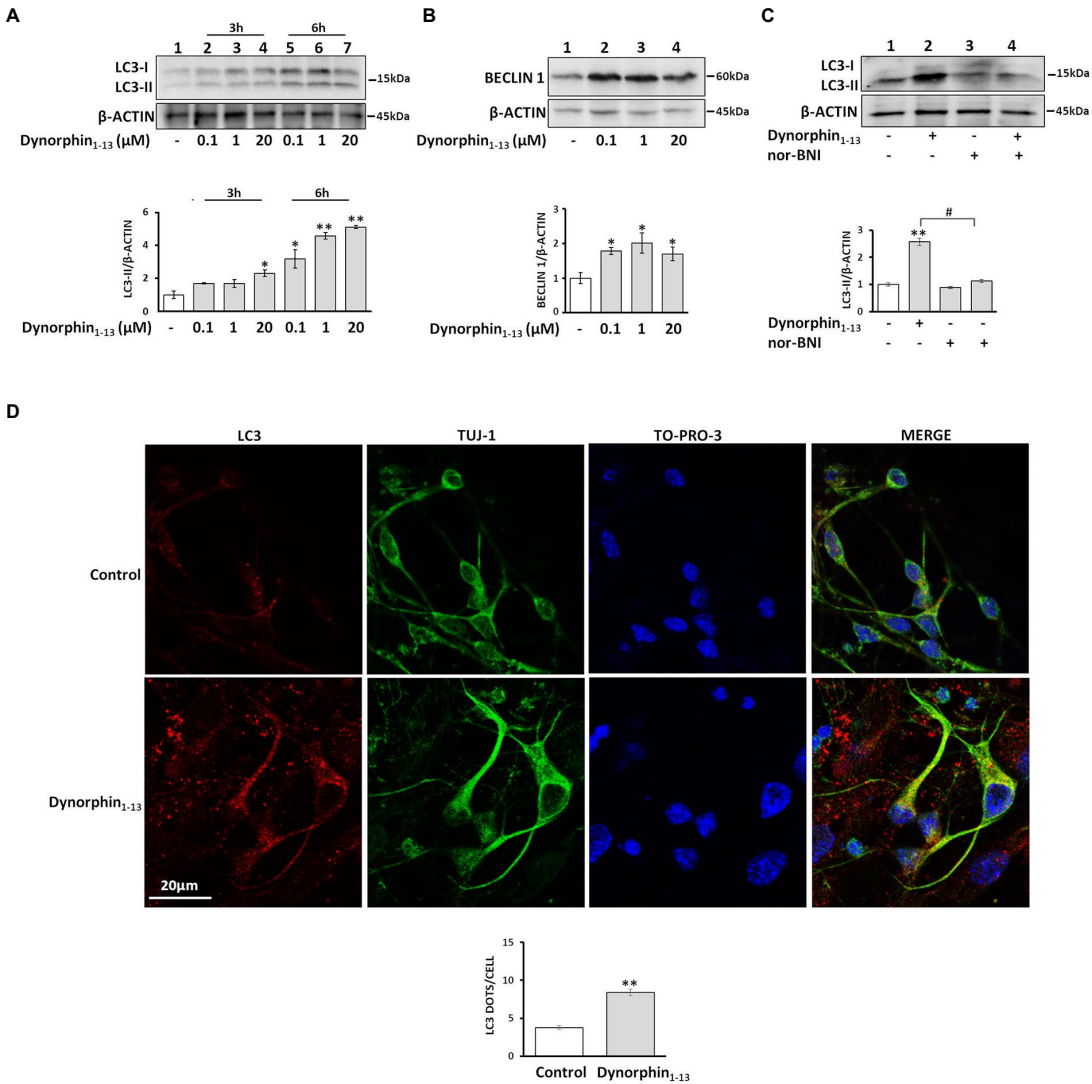
Dynorphin induces κ-ΟR-mediated autophagy in neuronal cells. Treatment of κ-Neuro-2A cells with dynorphin results in upregulation of LC3-II in a dose-and time-dependent manner. **(A,B)**. The levels of LC3-II **(A)** and Beclin1 **(B)** after dynorphin_1-13_ treatment for 6 h were detected by Western blotting and normalized to β-actin levels. Data represent mean ±SEM of three independent experiments; Statistical analysis was performed using one-way ANOVA. *p < 0.05 and **p < 0.01 as compared to untreated samples. **(C)** κ-Neuro-2A cells were pre-treated for 1 h, with or without the κ-ΟR antagonist nor-BNI (5 μM) following administration with 1 μΜ dynorphin for 6 h. The LC3-II levels were detected by Western blotting. β-actin served as loading control. Error bars represent mean ±SEM of three independent experiments. Statistical analysis was performed using two-way ANOVA, **p < 0.01 as compared with untreated samples, ^#^p < 0.05 as compared with samples in the presence of nor-BNI. **(D)** Representative confocal images of hippocampal embryonic DIV 10 neurons were treated or not with 1 μΜ dynorphin for 6 h and immunostained with antibodies against LC3B (1:200) and Tuj1 (1:1000) to label autophagosomes and mature dendrites, respectively, and the nuclear dye TO-PRO3 (1:500). Statistical analysis was performed using one-way ANOVA. **p < 0.01 as compared with untreated cells.

To deduce whether other κ-OR agonists exert similar effects on autophagy initiation, we exposed κ-Neuro-2A cells to varying concentrations of the endogenous κ-OR neuropeptide dynorphin_1-13_, which also resulted in increased LC3-II and Beclin 1 levels (Figures 2A,B). This effect was blocked by the selective κ-ΟR antagonist nor-BNI (Figure 2C). Finally, dynorphin_1-13_-treated primary neuronal cultures indicated an increase of LC3-positive puncta compared to control neurons (Figure 2D). These data suggest that κ-OR-induced autophagy is not selective to U50,488H, but can also be mediated by the endogenous κ-ΟR neuropeptide dynorphin.

**Figure 3:**
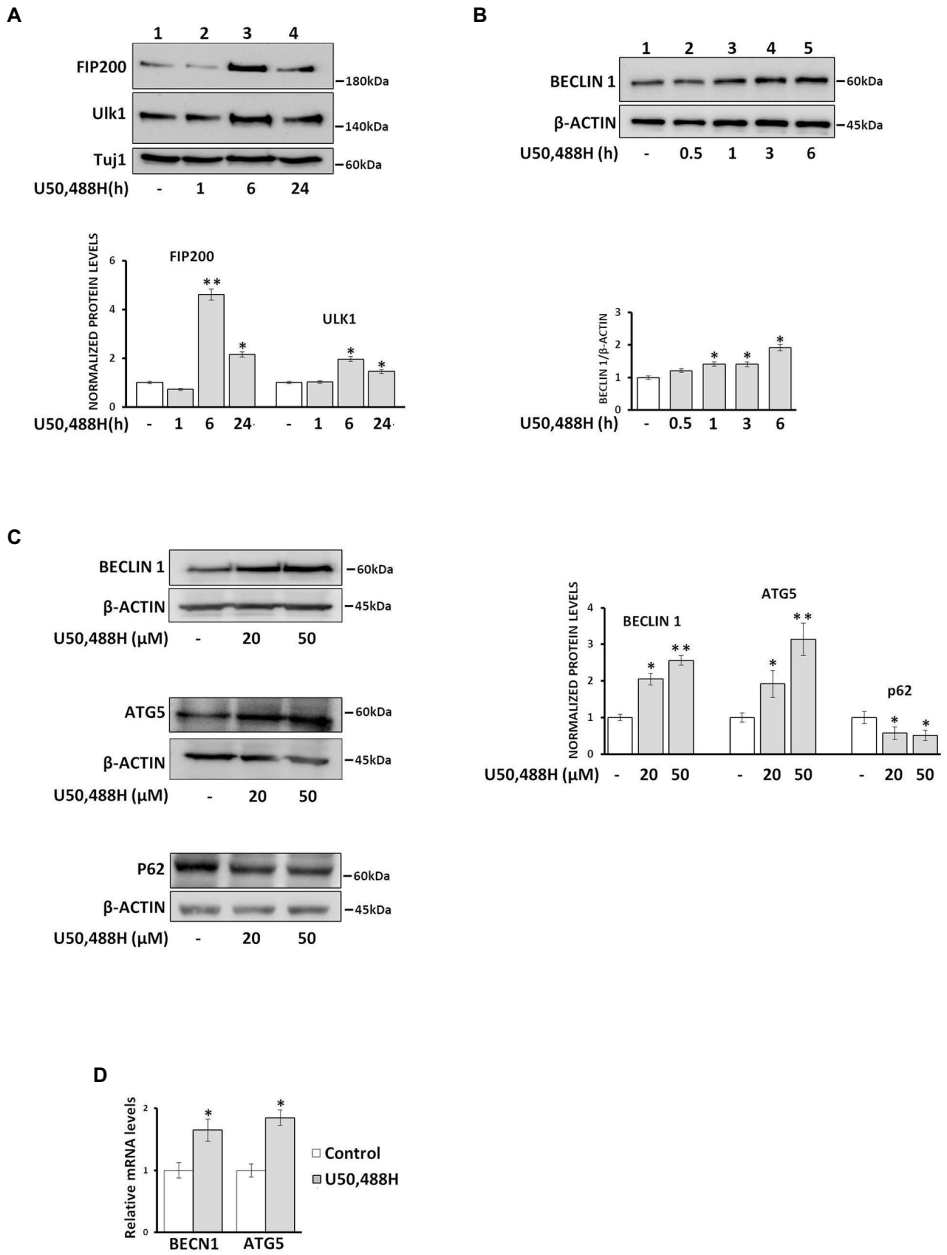
U50,488H treatment of neuronal cultures upregulates autophagosome biogenesis. **(A)** Primary neuronal cultures were treated with 20 μΜ U50,488H for the indicated time intervals and the levels of pre-autophagic markers FIP200 (1:1000) and Ulk1 (1:1000) were identified by western blotting using the corresponding antibodies. Tuj-1 protein levels were used as loading control. **(B)** Beclin 1 is upregulated in a time dependent manner in κ-Neuro-2A cell lysates upon 20 μΜ U50,488H administration. Beclin 1 levels were normalized to β-actin. **(C)** The levels of Beclin 1 and ATG5, were increased whereas those of p62 decreased in a dose dependent manner. The protein levels were normalized using β-actin. **(D)** κ-Neuro-2A cells were treated with 20 μΜ U50,488H for 6 h and the mRNA levels of Becn1 and Atg5 were examined by real-time PCR. Error bars represent average ± SΕΜ. Statistical analysis was performed using one way ANOVA. All experiments were performed at least three times. *p < 0.05 and **p < 0.01 as compared with untreated samples.

Because a key initial event of the autophagosome biogenesis is the formation of the pre-autophagosomal structure (PAS), composed of ULK1, which is a complex of a serine/threonine protein, with the focal adhesion kinase (FIP200) and other proteins, we examined the timing of U50,488H-mediated early autophagic events. Treatment of primary neuronal cultures with U50,488H for 1, 6, and 24 h, triggered a marked increase of FIP200 and ULK1 protein levels reaching a peak at 6 h agonist exposure (Figure 3A). In parallel, U50,488H treatment of κ-Neuro-2A cells for various time intervals indicated a time-dependent increase of Beclin1, a key mediator of autophagosome formation. This increase peaked at 6 h following U50,488H administration (Figure 3B). Similarly, as shown in Figure 3C, exposure of κ-Neuro-2A cells to U50,488H, increased the protein levels of ATG5 and Beclin 1, with a concomitant decrease of p62 known to increase when autophagy is inhibited and decrease when autophagy is induced (Bjorkoy et al., 2009). Additionally, *Becn1* and *Atg*5 mRNA levels were also elevated after 6 h U50,488H cell exposure (Figure 3D). These results clearly demonstrate that κ-OR is involved in autophagosome biogenesis in neuronal cells.

#### Identification of the κ-opioid receptor signaling pathway that regulates the autophagic machinery

**Figure 4:**
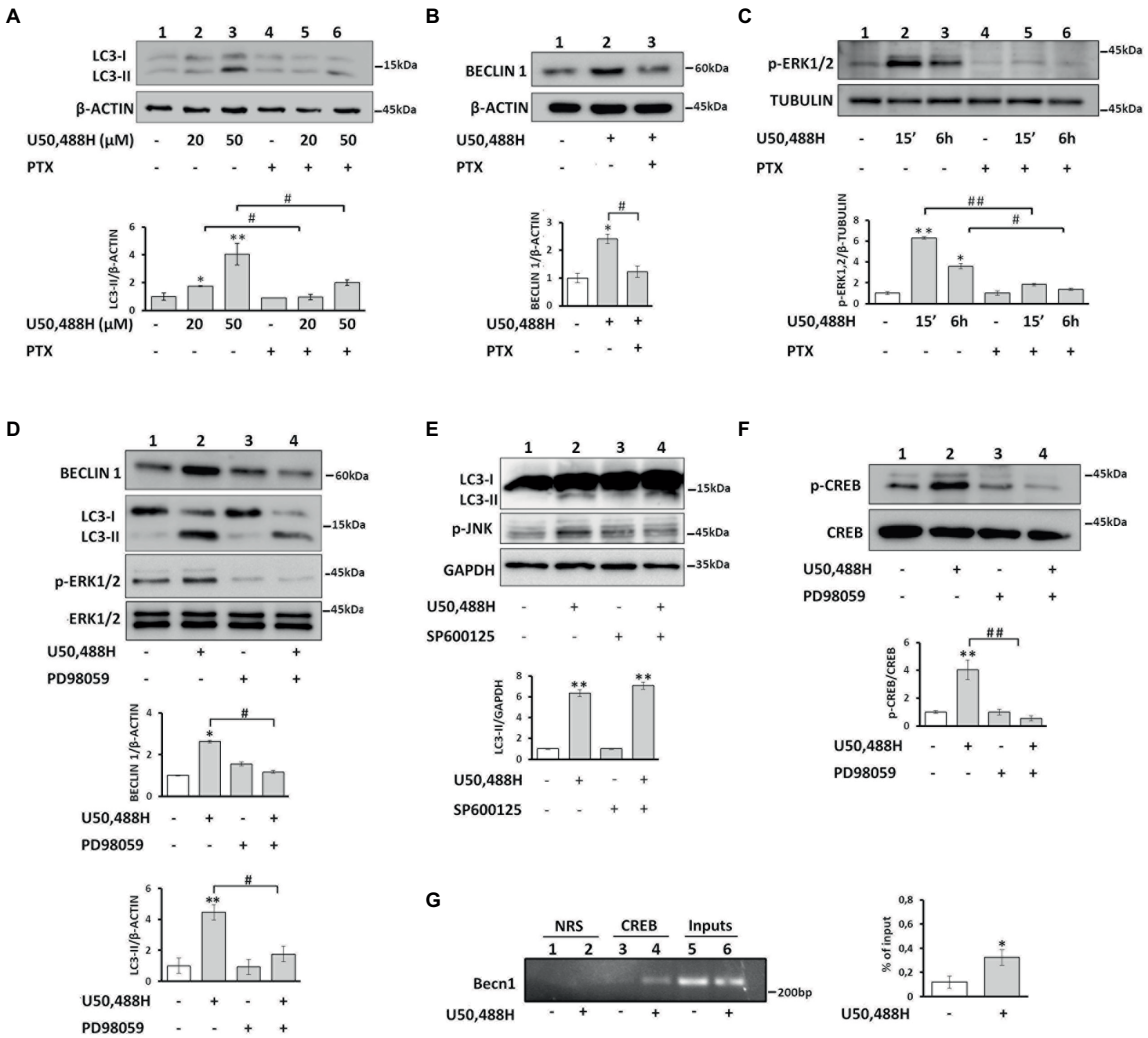
κ-OR activation induces autophagy in a G_i/o_ protein, ERK 1,2 kinase and p-CREB dependent manner. **(A)** κ-Neuro-2A cells were pre-treated with PTX (100 ng/mL) for 16 h, followed exposure to U50,488H for 6 h and LC3-II levels were assessed by Western immunoblotting. β-actin was used as loading control. Error bars represent mean values±SEM; Statistical analyses were performed using two-way ANOVA; *p < 0.05 and **p < 0.01 as compared to the untreated samples; ^#^*p* < 0.05 as compared to U50,488H treated samples. **(B)** Beclin 1 levels were detected after PTX pre-treatment following administration with 20 μΜ U50,488H for 6 h. β-actin was used as loading control. Error bars represent mean values±SEM; two-way ANOVA was performed. *p < 0.05 as compared to the untreated samples and ^#^*p* < 0.05 as compared to treated samples. **(C)** κ-Neuro2A cells were pre-treated with PTX for 16 h, prior to U50,488H (20 μΜ) for 15 min and 6 h exposure and the levels of p-ERK1,2 were quantified. Tubulin was used as loading control. Error bars represent mean values ± SEM. Two-way ANOVA was performed with *p < 0.05 and **p < 0.01 as compared to the untreated samples; ^#^*p* < 0.05 and ^##^*p* < 0.01 as compared to U50,488H treated samples. **(D)** κ-Neuro-2A cells were pre-treated with the ERK1,2 inhibitor PD98059 (20 μΜ) for 2 h, followed by U50,488H (20 μΜ) administration for 6 h. Total ERK1,2 was used for quantification. Error bars represent mean values±SEM. Statistical analyses were performed using two-way ANOVA. *p < 0.05 and **p < 0.01 as compared with untreated cells; ^#^*p* < 0.05 as compared with samples in the presence of U50,488H. **(E)** The levels of p-JNK were estimated in κ-Neuro-2A cells in the presence or not of the JNK inhibitor SP600125 (20 μΜ) for 30 min prior to 6 h U50,488H (20 μΜ) administration. All experiments were performed three times. GAPDH was used as loading control. Error bars represent mean values ± SEM. Statistical analyses were performed using two-way ANOVA. **p < 0.01 as compared with untreated cells. **(F)** κ-Neuro-2A cells were pre-treated with or without PD98059 (20 μΜ) for 2 h, followed by U50,488H (20 μΜ) exposure for 6 h. The phosphorylated levels of CREB were analyzed by Western blotting using an anti-p-CREB (Ser133) (1:500) serum and normalized to total CREB using a specific antibody (1:1000). Statistical analysis was performed using two-way ANOVA. **p < 0.01 as compared with untreated samples and ^##^*p* < 0.01 as compared with samples in the presence of U50,488H. **(G)** Chromatin immunoprecipitation was performed in κ-Neuro-2A cells as described in Materials and methods using an anti-CREB (10 μg) (lanes 3, 4) or normal rabbit serum (NRS) (lanes 1, 2). Immunoprecipitated Becn1 promoter region was amplified by PCR and the densitometry of the bands was quantified by Image J software on an agarose gel. CREB immunoprecipitated chromatin from control and U50,488H treated samples were normalized to their respective inputs. Error bars represent mean ± SEM of three independent experiments. Statistical analysis was performed using one-way ANOVA. *p < 0.05 as compared with untreated samples.

The κ-OR is coupled to pertussis toxin-sensitive Gi/o proteins to regulate a variety of effectors (Bruchas et al., 2010; Papakonstantinou et al., 2015). Το define the role of G proteins, we pretreated κ-Neuro-2A cells with pertussis toxin (PTX), which ADP-ribosylates Gαi/o subunits. PTX blocked U50,488H-mediated increase of LC3-II and Beclin 1 levels (Figures 4A,B), suggesting that Gi/o proteins are important players in κ-ΟR-mediated autophagy. To examine whether ERK1,2 is implicated in κ-OR-induction of autophagy the levels of ERK1,2 phosphorylation of κ-Neuro-2A cells were assessed in the presence or absence of PTX. U50,488H enhanced ERK1,2 phosphorylation after 15 min and 6 h post-exposure and this effect was abolished by PTX exposure (Figure 4C). Moreover, when the cells were pretreated with the ERK1,2 inhibitor PD98059 prior to agonist activation, U50,488H-mediated-ERK1,2 phosphorylation was abolished with a concomitant decrease of the LC3-II and Beclin1 levels, relative to the untreated cells (Figure 4D). We next wondered whether other members of MAP kinases are involved in κ-OR mediated autophagy. To elucidate that, we studied the implication of JNK kinase in κ-ΟR induced autophagy. As shown in Figure 4E, no effects on LC3-II accumulation were detected in U50,488H-treated cells relative to the untreated ones upon pre-treatment with the JNK inhibitor SP600125. These results suggest that ERK1,2 is implicated in κ-OR-mediated autophagy.

#### κ-opioid receptor regulates Beclin1 transcription via CREB activation

Because it is known that CREB regulates various autophagic genes (Seok et al., 2014) and that a consensus CRE binding site (TGACGTCA) exists in the mouse *Becn1* promoter, we sought to determine if Beclin 1, a fundamental protein in autophagic process is regulated by p-CREB upon U50,488H exposure. CREB was phosphorylated in response to U50,488H cell exposure, and this effect was abolished by the ERK1,2 inhibitor PD98059 (Figure 4F). Moreover, chromatin immunoprecipitation (ChIP) assay in isolated chromatin fragments of κ-Neuro-2A cells indicated that CREB binding in the *Becn1* promoter was greatly enhanced by U50,488H exposure relative to untreated cells (Figure 4G). Therefore, κ-ΟR-mediated increase in Beclin1 levels appears to involve transcriptional activation of the Beclin1 gene by ERK1,2-activated CREB.

#### U50,488H administration induces autophagy and promotes synaptic alterations in mouse hippocampus

**Figure 5:**
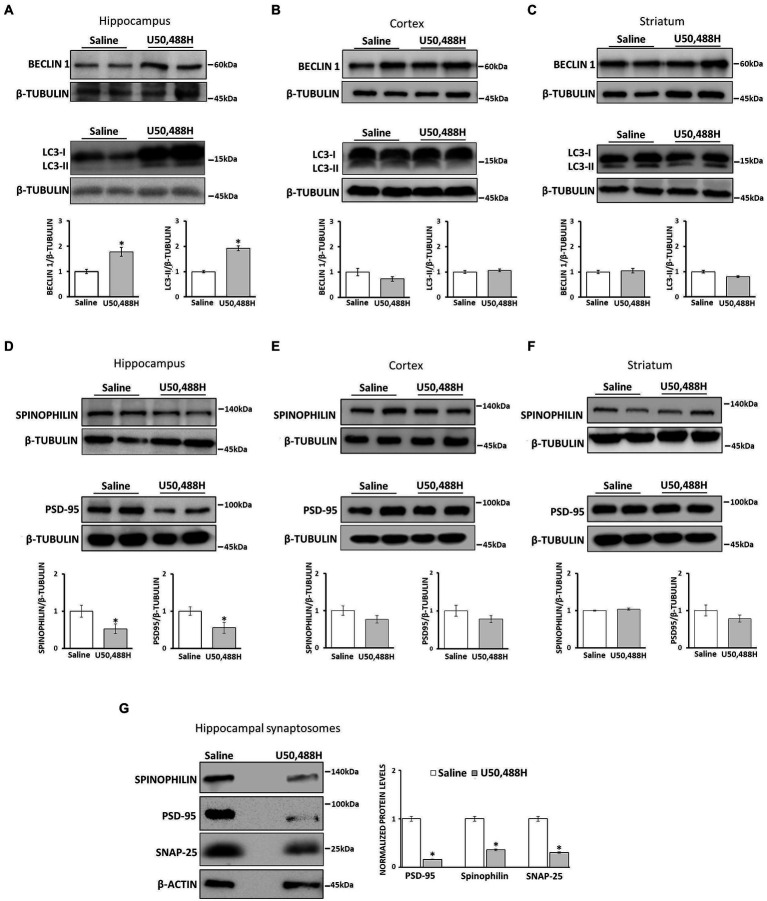
κ-ΟR activation induces autophagy in mouse hippocampus and alters synaptosomal protein levels. Male mice were injected i.p. with saline or 5 mg/kg U50,488H for six consecutive days and the hippocampus **(A),** cortex **(B)**, and striatum **(C)** were isolated and lysed as described in section “Materials and methods.” Autophagosome accumulation was measured by Western blotting using LC3B and Beclin 1 antibodies. Brain lysates from hippocampus **(D)**, cortex **(E)**, and striatum **(F)** of saline-and U50,488H-injected mice were isolated and the protein levels of spinophilin (1:1000) and PSD-95 (1:1000), were measured by immunoblotting using the corresponding antibodies. β-tubulin was used as loading control. **(G)** Hippocampal synaptosomes of saline-or U50,488H-injected mice were isolated and the levels of spinophilin, PSD-95, and SNAP25 (1:1000) were detected using the corresponding antibodies and quantified by Image J software using β-actin. All data are presented as average ± SEM from three independent experiments (*n* = 4/group, *p < 0.05 as compared with saline group using one-way ANOVA with *post-hoc* test).

We next sought to examine whether we could recapitulate U50,488H-mediated κ-ΟR autophagy *in vivo* and determine whether specific brain regions are involved. To this end, mice were injected with saline (vehicle) or U50,488H for 6 consecutive days and the levels of LC3-II and Beclin1 were measured in the hippocampus, cortex and striatum. U50,488H resulted in a significant increase of LC3-II and Beclin1 in the mouse hippocampus as compared to vehicle, but with no significant changes in cortical and striatal lysates (Figures 5A–C). Collectively, these results suggest that κ-OR-mediated autophagy is detected specifically to the mouse hippocampus.

Autophagy contributes to synaptic plasticity by degrading specific proteins that are essential for synaptic function and spine remodeling (Nikoletopoulou et al., 2017). To elucidate whether U50,488H-mediated autophagy leads to synaptic alterations, initially the levels of proteins enriched in dendritic spines such as PSD-95 and spinophilin, were examined in the hippocampus, cortex and striatum of U50,488H-treated mice. As shown in Figure 5D, spinophilin and PSD-95 in hippocampal lysates were significantly decreased in U50,488H-treated mice compared with saline-treated controls. However, no significant alterations for these proteins were detected in the cortex or striatum (Figures 5E,F). This was further confirmed in isolated synaptosomes where a pronounced decrease of spinophilin, PSD-95, as well as SNAP25 was detected in U50,488H-injected mice, as compared to control ones (Figure 5G). All these suggest that these synaptic proteins are degraded, possibly by being engulfed in the κ-ΟR-mediated autophagic cargo.

**Figure 6:**
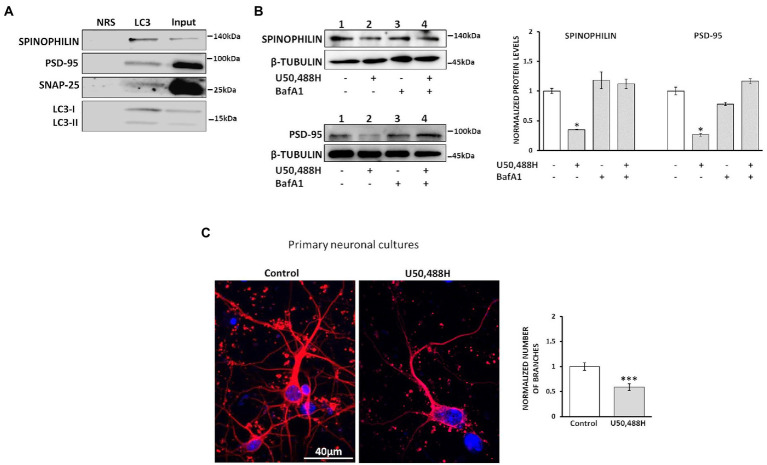
κ-ΟR-mediated autophagy leads to degradation of synaptosomal proteins. **(A)** Hippocampal lysates (800 μg) were immunoprecipitated with 2 μg of an LC3B antibody and immunoblotted with spinophilin, PSD-95, and SNAP25. NRS immunoprecipitated samples were used as negative control. The experiment was performed 3 times. **(B)** κ-Neuro-2A cells were pre-treated with 20 nM BafA1 for 24 h, following exposure with 20 μΜ U50,488H for 16 h. Cell lysates were subjected to 10% SDS-PAGE and the protein levels of spinophilin, PSD-95 and SNAP25 were detected using the corresponding antibodies. Quantification of the synaptic proteins was normalized using β-tubulin. Error bars represent mean values ± SEM of three independent experiments. Statistical analysis was performed using one-way ANOVA, *p < 0.05 as compared with the values in the absence of agonist. **(C)** Primary hippocampal neuronal cultures were treated for 24 h with 20 μΜ U50,488H and labeled with a Tuj1 antibody (1:1000). Nuclei were stained with TO-PRO-3 (blue) (1:500). Graph represents mean ± SEM of the number of branches calculated from 100 Tuj1-positive neurons of U50,488H-treated neurons normalized to control ones set as 1; scale bar: 40 μM. Statistical analysis was performed using one-way ANOVA, ***p < 0.001 as compared with the untreated samples.

To test this hypothesis and in view of the known interaction of LC3 with autophagic cargos through the LC3-interacting regions (LIR) of various proteins, spinophilin, SNAP25 and PSD-95 among them (Nikoletopoulou et al., 2017; Johansen and Lamark, 2020), we examined whether these proteins interact with LC3. Co-immunoprecipitation studies of hippocampal lysates using an LC3 antibody indicated that spinophilin, PSD-95 and SNAP25 do interact with LC3 (Figure 6A). Moreover, to further define whether these synaptic protein alterations are indeed due to autophagy induction we measured their levels in the presence of BafA1. Treatment of κ-Neuro-2A cells with U50,488H decreases the levels of spinophilin and PSD-95. Inhibition of autophagy by BafA1 treatment did not alter these protein levels, suggesting that U50,488H-κ-ΟR activation indeed leads to degradation of these synaptosomal proteins (Figure 6B). Finally, to verify whether these κ-OR-mediated effects are due to alterations in neuronal sprouting, the number of branches in U50,488H-treated hippocampal neuronal cultures were measured. U50,488H significantly reduced the number of branches relative to the controls (Figure 6C), suggesting that κ-ΟR-induced autophagy modulates neuronal sprouting, possibly by degrading key synaptic proteins.

#### Activation of the endogenous κ-opioid receptor/dynorphin system upon stress upregulates autophagy in the hippocampus and results in synaptic alterations

**Figure 7:**
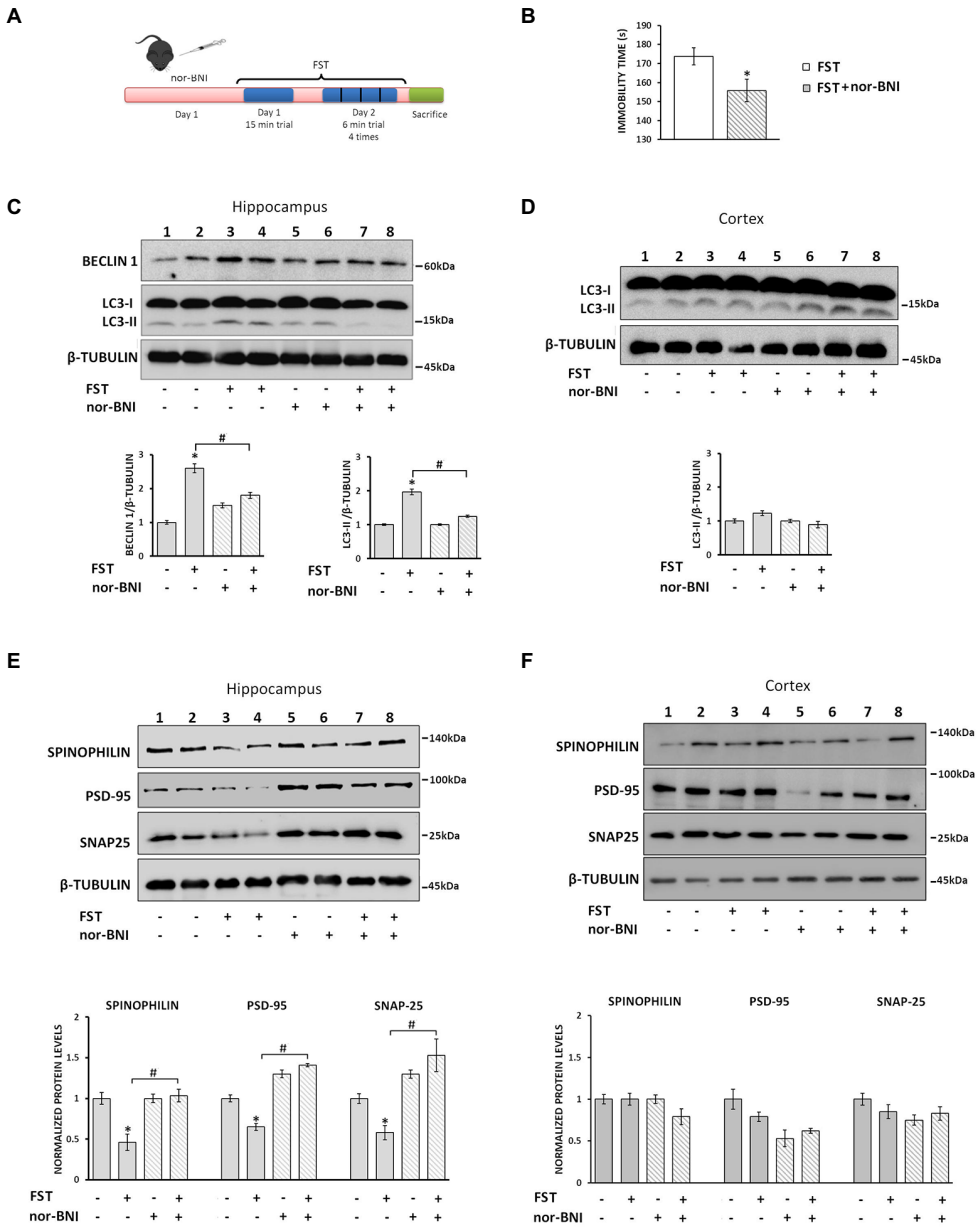
Nor-BNI blocks FST-induced autophagy promoting synaptic alterations in mouse hippocampus. **(A)** Experimental timeline of the nor-BNI administration protocol and the 2-day repeated FST in C57BL/6 J mice. Four groups of animals [saline and nor-BNI (non-stressed groups), and saline and nor-BNI exposed to FST (stressed groups)], (*n* = 6/group) were injected i.p. with saline or the specific κ-OR antagonist nor-BNI (10 mg/kg) on day 1. One hour after injection, mice were subjected to 15 min FST. On the second day, mice were subjected to 4×6 min FST trials and immediately sacrificed and hippocampi and cortices were isolated. **(B)** For behavioral analysis the immobility time of mice was quantified using a video tracking software as described in Materials and Methods. Statistical analysis was performed by one-way ANOVA variance, *p < 0.05 relative to FST saline group. For autophagy induction, LC3-II and Beclin 1 levels were measured in hippocampus **(C)** and cortex **(D)** by Western immunoblotting. **(E)** The levels of spinophilin, PSD-95 and SNAP25 in the hippocampus were detected from the four groups to evaluate differences between the stressed and non-stressed animals injected with saline or nor-BNI. β-tubulin was used as the loading control. **(F)** Spinophilin, PSD-95 and SNAP25 levels were detected from isolated cortical lysates (50 μg) from the four mice groups. The ratio of proteins to β-tubulin was normalized to control (saline non-stressed), which is set as 1. All data are presented as average ± SEM (*n* = 6/group, *p < 0.05 as compared with the saline non-stressed group and ^#^*p* < 0.05 compared with the saline stressed ones using two-way ANOVA with *post-hoc* test).

It is well documented that the κ-ΟR/dynorphin system plays an important role in anxiety and stress-related behaviors and that κ-OR antagonists exhibit anxiolytic effects (McLaughlin et al., 2003; Fava et al., 2016). To examine whether stress-induced endogenous dynorphin release impacts on autophagy regulation, we examined the consequences of acute stress on autophagy in the hippocampus. To this end, mice injected with either vehicle or the κ-OR selective antagonist, nor-BNI, which is known to exert anxiolytic effects, were subjected to a two-day modified forced swim test (FST) (Figure 7A). Male C57BL/6 J mice were divided into 4 groups saline-control or saline-FST (stressed) and norBNI-not stressed or nor-BNI-FST (stressed). nor-BNI significantly decreased immobility time following the FST, compared to saline-treated mice, suggesting that nor-BNI attenuates stress-related behavior (Figure 7B). Subsequently, the levels of the autophagic markers LC3-II and Beclin1 were measured in isolated hippocampal lysates and found to be significantly increased in stressed animals (FST) relative to vehicle injected-non-stressed ones (Figure 7C). In contrast, this increase in autophagic markers was not detected in nor-BNI-treated mice under control or FST conditions when compared with the saline-control group (Figure 7C). Moreover, as expected, no significant alterations of LC3-II levels were detected in the cortices of the same treatment groups (Figure 7D), confirming that the hippocampus is the target region for κ-ΟR-induced autophagy under acute stress.

Finally, to confirm that dynorphin/κ-OR-induced autophagy-mediated changes in hippocampal synapses during stress may be rescued by nor-BNI, we measured the levels of synaptic proteins in hippocampi of stressed and naïve animals subjected to nor-BNI, or saline treatment. Our results demonstrated that spinophilin, PSD-95, and SNAP25 protein levels were significantly reduced in stressed animals relative to the control ones (Figure 7E). In contrast, the levels of these synaptic proteins in nor-BNI injected mice prior to FST were at the same levels as the control nor-BNI-injected ones devote of the stressor (Figure 7E, lanes 5–8). Again, no significant alterations in cortical lysates of these proteins were detected, irrespective of the stress-related regime or nor-BNI administration status (Figure 7F). Collectively, these results suggest that the endogenous dynorphin release due to the acute FST results in κ-ΟR-mediated induction of autophagy that in turn leads to hippocampal synaptic alterations.

## Discussion

**Figure 8:**
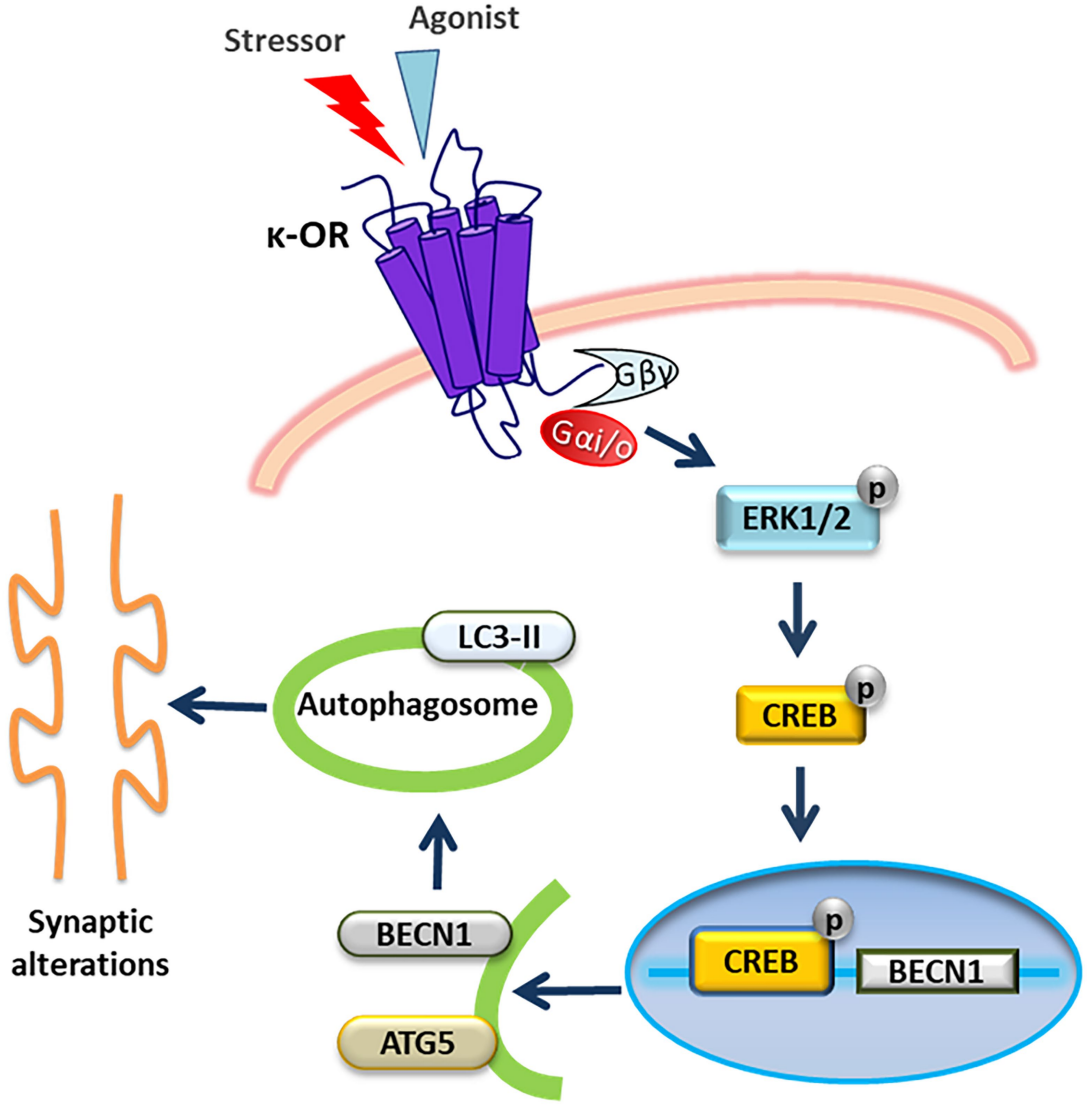
Schematic representation of a putative signaling pathway via which κ-OR activation triggers autophagy resulting in synaptic alterations. Activation of κ-ΟR in neuronal cells leads to ERK1,2 phosphorylation mediated by Gi/ο proteins. Αctivated ERK1,2 subsequently phosphorylates CREB which in turn translocates to the nucleus to activate *Becn1* gene expression. Upregulation of Beclin1 and Atg5 promotes the initiation of autophagy resulting in alterations of hippocampal synaptic proteins enriched in dendritic spines.

Previous studies have shown that in the brain the κ-OR is involved in motivation, stress-related responses and adult neurogenesis and that κ-ΟR agonists and antagonists exert potent pro-and anti-depressant effects, respectively, in rodents (Lutz and Kieffer, 2013; Kibaly et al., 2019). However, the mechanistic details of the aberrant synaptic function and resulting behavior mediated by κ-OR upon stress remain elusive. In the present study, we demonstrate that κ-OR plays a role in stress-induced autophagy, which leads to synaptic alterations. κ-ΟR-induced autophagy occurs primarily in the hippocampus despite κ-ΟR’s high expression levels in cortex and striatum. Gi/o proteins and U50,488H-induced ERK1,2 activation are responsible for κ-OR-mediated autophagy. An intriguing finding of the present study has been that U50,488H-dependent-CREB activation regulates the transcription of the *becn1* gene, confirming that κ-ΟR activation leads to transcriptional induction of specific autophagic genes. We are thus proposing a putative G protein dependent signaling pathway for the control of autophagy by κ-ΟR. Activated κ-OR leads to activation of ERK1,2 which phosphorylates CREB, with the later promoting alterations of autophagic genes leading to synaptic protein changes (Figure 8). This proposal is in agreement with previous observations which have shown that heterotrimeric G proteins control autophagic sequestration in HT-29 cells (Ogier-Denis et al., 1995, 1996), and that Gαi3, which is activated by κ-OR (Papakonstantinou et al., 2015), plays a crucial role in autophagosomal membrane compartmentalization (Gohla et al., 2007) and autophagy initiation (Gotthardt et al., 2006). It is also compatible with previous findings suggesting that a dynamic interplay between Gαi3, the activator of G-protein signaling 3 and Gα-interacting vesicle-associated protein (GIV), are signaling components that determine whether autophagy is induced or inhibited (Garcia-Marcos et al., 2011), and that Gαi3 interacts with RGS4 to increase autophagy (Bastin et al., 2020).

A number of different GPCRs have been shown to regulate autophagy albeit through different mechanisms (Wauson et al., 2014). Thus, dopamine D2 and D3 receptors were shown to be positive regulators of autophagy, involving the Akt–mTOR and AMP-activated protein kinase (AMPK) signaling pathways (Wang et al., 2018). Similarly, activation of the β2-adrenergic receptor upregulates autophagy and increases collagen degradation in order to maintain cardiac extracellular matrix homeostasis (Aranguiz-Urroz et al., 2011). Methamphetamine exposure also induces autophagy via the κ-OR as a pro-survival response against apoptotic endothelial cell death, an effect that is also mediated by ERK1,2 activation and inactivation of the Akt/mTOR pathway (Ma et al., 2014). In the mouse hippocampus, acute or chronic morphine administration upregulates autophagic flux as protective response towards morphine-induced neuronal death and consequent spatial memory deficits (Pan et al., 2017). Moreover, chronic morphine administration alters synaptic plasticity in the hippocampus and results in spine and excitatory synapse density reduction, via generation of reactive oxygen species leading to Endoplasmic Reticulum (ER) stress activation (Cai et al., 2016). Finally, it was noted that morphine-mediated autophagy involves activation of ER stress with subsequent downstream astrocyte activation via the μ-opioid receptor (Sil et al., 2018), a finding confirming that opioids are indeed potential positive regulators of autophagy.

The immediate responses to stressful stimuli include neuro-morphological changes in multiple brain areas including the hippocampus (Leuner and Shors, 2013). Acute stress reduces the density of dendritic spines, alters the location of postsynaptic elements of excitatory synapses, and impairs long-term potentiation and memory (Leuner and Shors, 2013; Shen et al., 2015). Chronic stress has been reported to enhance autophagy in rodents (Xiao et al., 2018; Woo et al., 2020). Furthermore, a role for autophagy in depressive-like behaviors and cognitive impairment has been demonstrated following prenatal stress (Zhang et al., 2017). Our current findings extend the existing evidence by demonstrating a plausible scenario where the dynorphin/κ-ΟR system initiates autophagy, which leads to stress-induced synaptic alterations.

Neuronal autophagy plays a major role in brain function by modulating synaptic organization and morphogenesis (Hernandez et al., 2012; Nikoletopoulou et al., 2017). It contributes to synaptic plasticity by degrading specific synaptic proteins such as PSD-95, PICK1 and SHANK3, which play important roles in synaptic function and spine modeling. This implies a direct link between autophagy and pruning of synaptic connections during postnatal development (Nikoletopoulou et al., 2017). In agreement with these predictions, our findings demonstrate that U50,488H-κ-ΟR activation of primary hippocampal cultures reduces the number of neurite branches. In addition, sub-chronic U50,488H administration in mice led to degradation of the key scaffolding synaptic proteins, spinophilin, PSD-95 and SNAP25, particularly in the hippocampus, but not in the cortex or striatum. We specifically chose to examine these proteins as they are implicated in dendritic spine remodeling. Spinophilin localizes in the postsynaptic compartment, is enriched in dendritic spines, and modulates spine morphogenesis and maturation through the regulation of the actin cytoskeleton (Feng et al., 2000). It also interacts directly with opioid receptors and other GPCRs to regulate their trafficking and signaling that leads to synaptic alterations (Feng et al., 2000; Sarrouilhe et al., 2006; Fourla et al., 2012). On the other hand, SNAP25 plays a crucial role pre-synaptically by mediating synaptic vesicle fusion. Of note is that SNAP-25 and PSD-95 are substrates of autophagic degradation modulating dendritic spine morphology and function (Kallergi and Nikoletopoulou, 2021).

Stress blocks long term potentiation through release of endogenous opioids including the release of the endogenous κ-opioid neuropeptide dynorphin. Activation of κ-ΟR *in vivo* promotes aversion, dysphoria, depression, and anxiety-like behaviors (Tejeda et al., 2012; Lutz and Kieffer, 2013; Lalanne et al., 2014; Hang et al., 2015). Conversely, κ-OR antagonists prevent many effects of stress and counteract stress-induced behavioral responses and for this reason, are considered as novel therapeutics for stress-related disorders (Rorick-Kehn et al., 2014; Jacobson et al., 2020). Forced swim stress in rats elevates dynorphin A levels in the hippocampus (Shirayama et al., 2004), while chronic autophagy deficiency in dopamine neurons results in increased size of axon profiles, increased evoked dopamine release and rapid presynaptic recovery (Hernandez et al., 2012). Another interesting finding of the present study has been that FST in mice promoted autophagy, as indicated by the elevated levels of autophagic markers and this effect was prevented by administration of the κ-OR antagonist, nor-BNI. This suggests that the endogenous dynorphin/κ-OR system is involved in stress-induced autophagy and could be part of the orchestration of synaptic changes observed in the hippocampus under stress exposure. Interestingly, a concomitant decrease of the three synaptic proteins, spinophilin, PSD-95 and SNAP25, was also detected in the hippocampus but not the cortex of stressed animals. A slight increase on the levels of PSD-95 and SNAP25 in hippocampus detected in the nor-BNI-non-stressed mice could be attributed to the upregulation of BDNF observed by nor-BNI administration which regulates spinal density and morphology and found to increase the levels of synaptosomal proteins (Russo-Neustadt et al., 2004; Zhang et al., 2007; Ji et al., 2010; Kellner et al., 2014).

Based on our observations we postulate that degradation of the synaptic proteins spinophilin, SNAP25 and PSD95 could be attributed to their engulfment in the autophagosome. These findings are further supported by the observation that LC3 interacts with these three key protein substrates that possess putative LIR motifs in their sequence. In turn, this suggests that under acute stress, the release of dynorphin triggers the autophagic machinery leading to synaptosomal alterations in the hippocampus. It is interesting to note that similar synaptosomal alterations that are crucial for dendritic spine remodeling and are caused by autophagic degradation have also been reported previously under conditions of nutritional stress, through a BDNF-regulated mechanism (Nikoletopoulou et al., 2017). Autophagy was also reported to play a crucial role in postnatal spine pruning in layer V pyramidal neurons (Tang et al., 2014), suggesting that it plays a significant role in synaptic organization and morphogenesis.

Based on the present findings, we conclude that an interplay exists between κ-ΟR-mediated autophagy and stress-mediated synaptosomal alterations. Indeed, we propose the existence of a signaling pathway (Figure 8) correlating κ-OR-induced autophagy in neurons with synaptic hippocampal alterations under stress conditions. This κ-OR-mediated autophagy mechanism could possibly result in synaptic dysfunction in hippocampus that may contribute to the cognitive changes observed upon stress exposure. Given that κ-OR antagonists (Domi et al., 2018; Jacobson et al., 2020) are in phase II clinical trials for stress-related mood and anxiety disorders, it would be interesting to explore whether these drugs effectively alleviate stress-related pathologies via κ-OR-mediated-autophagy.

## Data availability statement

The original contributions presented in the study are included in the article, further inquiries can be directed to the corresponding author.

## Ethics statement

The animal study was reviewed and approved by the Ethics Committee of the National Centre for Scientific Research “Demokritos.”

## Author contributions

CK designed and performed the experiments, analyzed the data, and contributed to manuscript writing. ASo, ASy, and DP-D performed the experiments. AP and CK performed the behavioral studies. VN contributed to the data research. ZG supervised the study, analyzed data, and wrote the manuscript. All authors contributed to the article and approved the submitted version.

## Funding

This work was supported by the General Secretariat of Research and Technology (GSRT) grant Excellence NO-ALGOS-3722 to ZG. We also acknowledge the support by the OPENSCREEN-GR An Open-Access Research Infrastructure of Chemical Biology and Target-Based Screening Technologies for Human and Animal Health, Agriculture and the Environment (MIS 5002691), which is implemented under the Action “Reinforcement of the Research and Innovation Infrastructure,” funded by the Operational Program “Competitiveness, Entrepreneurship and Innovation” (NSRF 2014–2020) and co-financed by Greece and the European Union (European Regional Development Fund). ZG, CK, and ASy are members of the European COST Action CA18133, “ERNEST” a European Research Network on Signal Transduction and were supported to present the data.

## Conflict of interest

The authors declare that the research was conducted in the absence of any commercial or financial relationships that could be construed as a potential conflict of interest.

## Publisher’s note

All claims expressed in this article are solely those of the authors and do not necessarily represent those of their affiliated organizations, or those of the publisher, the editors and the reviewers. Any product that may be evaluated in this article, or claim that may be made by its manufacturer, is not guaranteed or endorsed by the publisher.

## Abbreviations

AC: Adenylyl cyclase
AMPK: AMP-activated protein kinase
ATG5: Autophagy related gene 5
AMPA: α-amino-3-hydroxy-5-methyl-4-isoxazolepropionic acid
ChIP: Chromatin immunoprecipitation
CREB: cAMP response element binding protein
DMEM: Dulbecco’s modified Eagle’s medium
ERK1,2: Extracellular signal-regulated protein kinase 1,2
FST: Forced swim test
GABA: γ-aminobutyric acid
GPCRs: G protein coupled receptors
HRP: Horseradish peroxidase
LC3: Microtubule-associated protein light chain
MAPK: Mitogen activated protein kinase
mTOR: Mechanistic target of rapamycin
nor-BNI: Nor-binaltorphimine
NRS: Normal rabbit serum
ORs: Opioid receptors
PBS: Phosphate-buffered saline
PSD-95: Postsynaptic density protein 95
PTX: *Bordetella pertussis* toxin
PVDF: Polyvinylidene difluoride
SDS-PAGE: Sodium dodecyl sulfate polyacrylamide gel electrophoresis
SNAP25: Synaptosomal associated protein 25
U50,488H: 2-(3,4-dichlorophenyl)-N-methyl-N-[(1R,2R)-2-pyrrolidin-1-ylcyclohexyl] acetamide
